# Metagenomic research on the structural difference of plaque microbiome from different caries stages and the construction of a caries diagnostic model

**DOI:** 10.1128/msystems.00044-25

**Published:** 2025-09-10

**Authors:** Lei Dong, Jiazhen Yang, Hui Wu, Yanfei Sun, Jiang Liu, Hao Yuan, Mingchao Wang, Yajie Dai, Fei Teng, Gongchao Jing, Fang Yang

**Affiliations:** 1 Department of Stomatology, Qingdao Women and Children's Hospital Affiliated to Qingdao University, Qingdao, Shandong, China; 2 Qingdao Huangdao Central Hospital 609297 , Qingdao, Shandong, China; 3 Qingdao Stomatological Hospital Affiliated to Qingdao University 678502, Qingdao, Shandong, China; 4 Qingdao Municipal Hospital, Qingdao, Shandong, China; 5 Qingdao OE Biotechnology Company Limited, Qingdao, Shandong, China; 6 Qingdao Institute of BioEnergy and Bioprocess Technology Chinese Academy of Sciences, Qingdao, Shandong, China; 7 Qingdao University of Science and Technology 66280, Qingdao, Shandong, China; University of California, San Francisco, San Francisco, California, USA

**Keywords:** dentin caries, microbiome, 2bRAD sequencing for microbiome, diagnostic model, different caries stages

## Abstract

Development of dental caries is a dynamic process; yet, there is limited knowledge on microbial differences at various stages of caries at higher resolution. To investigate the shifting microbiome profiles across different caries stages, 30 children were enrolled in this study, including 15 caries-active patients and 15 caries-free individuals. Plaque samples were collected from the buccal surface of caries-free subjects, defined as confident health (CH; *n* = 15). For caries-active individuals, plaque samples were collected from non-cavitated surfaces (defined as relative health [RH], *n* = 15), enamel caries (EC; *n* = 15), and dentin caries samples (DC; *n* = 15). All the above samples were sequenced through the 2bRAD sequencing platform to reveal the microbial community structures in each group. We identified significant differences in microbial community structures from different caries stages. First, the CH group showed the highest species richness (*P* < 0.05), and then followed by the RH and EC groups with lower richness, and the lowest richness was found in the DC group, yet no significant difference was found among the last three groups (*P* > 0.05). Second, the microbial structure exhibited the greatest difference between CH and DC groups, followed by the distance between RH/EC and DC groups, and the smallest difference was found between RH and EC groups. Third, specific species were found with a significant difference during the different caries stages. Therefore, we developed a diagnostic model using deep learning methods based on neural networks to diagnose different caries stages with an AUC of over 98%. This may provide methodological support for the understanding of the etiological factor in the pathological progression of dental caries.

**IMPORTANCE:**

The diagnosis and treatment of dental caries are crucial for human oral health. Previous studies have focused on the microbial differences between caries and healthy teeth, but there was not enough knowledge on the microbial differences at different stages of dental caries. Our findings could provide a high-resolution understanding of the microbial divergencies among different stages of dental caries and thus build microbial-based diagnostic models for differentiating dental caries status using deep learning methods with an accuracy of over 98%. This may provide methodological support for the understanding of the etiological factor in the pathological progression of dental caries.

## INTRODUCTION

Dental caries has always been an infectious disease that troubles people of all age groups in both developed and developing countries worldwide (1). Currently, due to the intake of high-precision sugars, the number of sufferers has been continually rising. The constant progression of dental caries can lead to the destruction of the tooth surface or even the loss of tooth structure, resulting in infection and pain. Once it begins, dental caries is irreversible, with treatment options limited to mechanical or surgical methods. Therefore, the prevention and diagnosis of dental caries is of critical importance, as it greatly impacts the maintenance of oral health.

Although numerous factors contribute to the development of dental caries, the prevailing consensus points to the microbial factors associated with plaque, specifically the imbalance between demineralization and remineralization of dental hard tissues caused by acids from microbial fermentation of dietary carbohydrates (2). The “ecological plaque hypothesis” (EPH) (3) states that the formation of dental plaque in a healthy individual follows an organized pattern of colonization by a range of bacteria that establishes a dynamic balance. Contrarily, disease is the result of an imbalance in the total microflora due to ecological stress, resulting in an enrichment of some “oral pathogens” or disease-related micro-organisms (3). Once the balance has been broken down, microbial imbalances can lead to the outgrowth of pathogenic bacteria (3–5).

With the advent of high-throughput sequencing methods, numerous researchers have focused on disclosing the microbial community imbalance in saliva and dental plaque between extreme populations, including caries-active and healthy individuals (6–13), aiming to explore the microbial differences associated with disease. However, the caries disease of those caries-active subjects was in different stages and with distinct genetic backgrounds; thus, the resulting caries-related biological risk factors could not be co-validated by different research groups (6–8, 10, 11, 13). Given that the development of dental caries is a gradual process, a detailed understanding of the microbiome at different stages of caries is essential, as it will yield more in-depth insights into the microbial etiology underlying dental caries. Metagenomics and amplicon sequencing (16S rRNA and ITS gene amplicon) have identified a diverse range of microbiomes associated with caries progression (14–21). Nevertheless, prior research has encountered considerable limitations due to technological constraints. Metagenomic sequencing exhibits lower resolution when analyzing low-biomass and high-host-contaminated samples. Although this technology offers relatively comprehensive microbial information, the data processing remains complex and costly. Amplicon sequencing can avoid the above drawbacks; however, its resolution is constricted to the genus level, which may result in the inability to accurately validate previous research findings across different studies. Therefore, limitations in sequencing resolution (restricted to the genus level) and individual heterogeneity have hindered the validation of those biomarkers in the microbial communities identified in these studies.

To overcome the aforementioned shortcomings and substantially enhance the efficiency of microbial research on dental caries, it is essential to develop a more effective sequencing method with high sensitivity. The “2bRAD sequencing for microbiome” (2bRAD-M) method, as a new technology, can generate species-level taxonomic profiles for microbiome samples that are low-biomass, high-host-contaminated, and degraded (22). It has been used to detect the microbial composition found on the kindergarten students’ palms and indoor sites, overcoming the challenges associated with low biomass and enhancing taxa resolution to the species level (23). This new technology allows for the identification of bacteria, fungi, and archaea even under low-biomass conditions. Qualitative analysis, such as estimating the abundance of microbial species, is robust to host DNA interference, greatly reducing the false-positive rate of the samples (22). Faced with host-gene-rich samples such as dental plaque or saliva, 2bRAD-M can overcome high host contamination by selecting appropriate primers and restriction enzymes. Consequently, it is well-suited for tracking changes in the complex microbiota during the progression of caries.

In this study, we aim to study the difference in the bacterial communities involved in different caries stages based on 2bRAD-M and to construct a precise diagnostic model for different stages through deep learning methods. Our results will help to determine the dynamic changes in microbial communities during the caries process. This will provide a new approach with innovative guiding significance for clinical prevention, diagnosis, and treatment of dental caries.

## RESULTS

### The comparison of microbial diversity at various caries stages

In this study, 60 dental plaque samples were subjected to 2bRAD-M sequencing and included for analysis, including confident health (CH), relative health (RH), enamel caries (EC), and dentin caries (DC). By categorizing organisms at different taxonomic levels, we identified a total of 41 phyla, 74 classes, 178 orders, 341 families, 1,019 genera, and 3,251 species, which are classified into three main domains: archaea, bacteria, and fungi. From the sequencing of 60 samples, we found 987 genera and 3,212 species in the bacterial kingdom; 4 genera and 4 species in the archaeal kingdom; and 28 genera and 35 species in the fungal kingdom.

**Fig 1:**
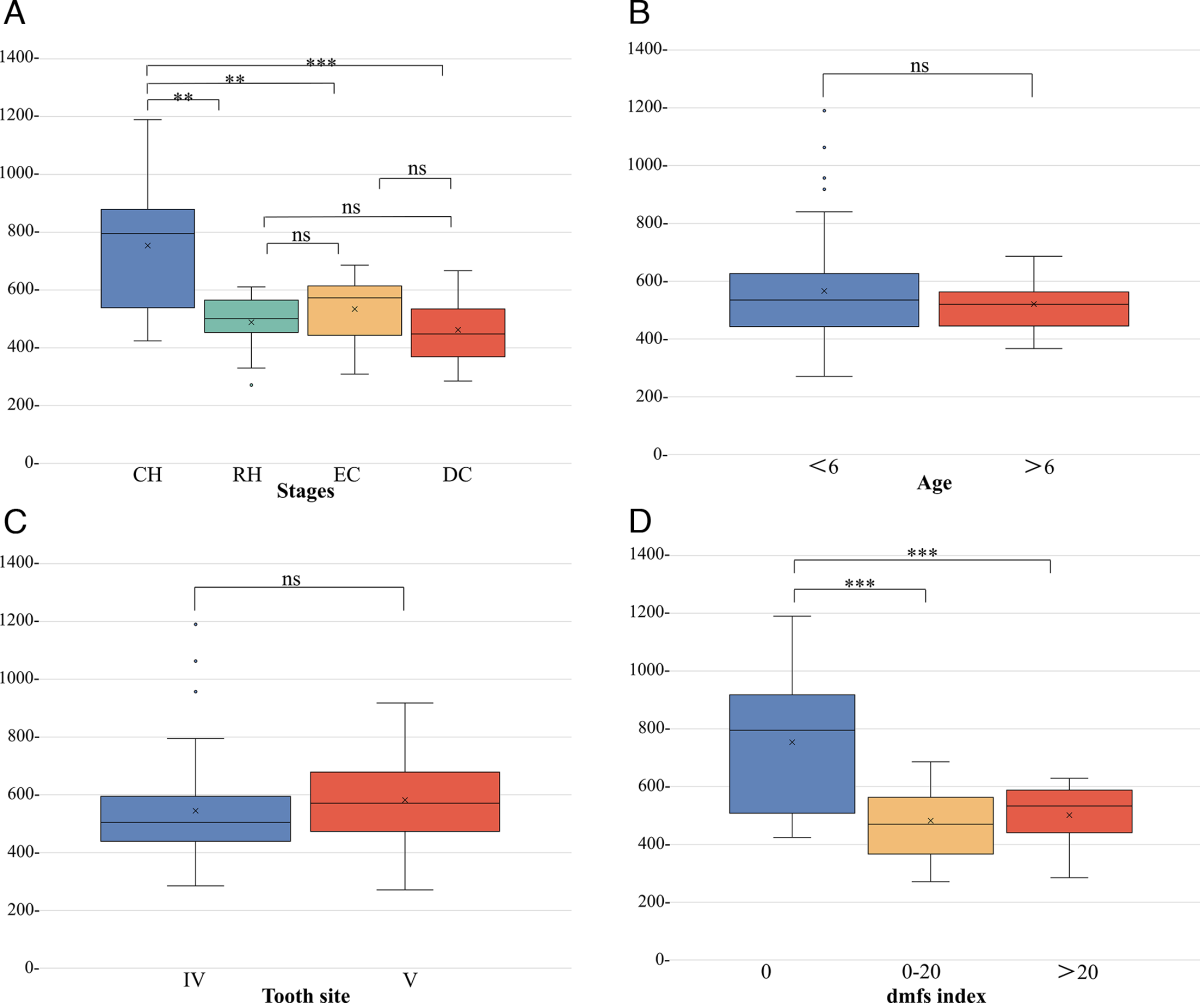
Microbial α-diversity evaluated by Chao1 index. (**A**) Boxplot of species diversity according to different stages. (**B** and **C**) Boxplot of species diversity according to age and tooth. (**D**) Boxplot of species diversity according to dmfs index.

We evaluated the effect of group assignment on microbial diversity. The comparisons of alpha diversity indices (Chao1 index) of the plaque microbiota were significantly different among the four groups. The Chao1 index of the CH group was the highest (*P* < 0.05), and the RH and EC groups had lower richness, followed by the DC group with the lowest richness, yet no significant difference was found among the last three groups (*P* > 0.05) (Fig. 1A). It was worthwhile noting that the “dmfs index” had a dominating effect on defining microbial diversity (*P* < 0.05) (Fig. 1D), while other factors (“age” and “tooth site”) had no significant effect (*P* > 0.05) (Fig. 1B and C). And we found no significant differences in species diversity among individuals (Fig. S1). Furthermore, the comparison of the effect sizes of host factors showed that the dmfs index is the host factor with the most profound impact on defining species diversity (Table 1). We conducted a permutational multivariate analysis of variance (PERMANOVA) using these metadata. Our results demonstrated that the grouping method for “different stages of caries” exhibits the highest *R*-squared, followed by the “dmfs index,” which aligns with the effect size figures mentioned above (Table S1).

**TABLE 1:** Effect size differences (Cohen’s *d*) between groups^*a*^

Parameter	Age	Tooth site	dmfs index
Cohen’s *d*	0.28	0.20	0.96
Effect size	Small	Small	Large

^*a*^ <0.02 = trivial; 0.2–0.5 = small; 0.5–0.8 = moderate; >0.8 = large.

### The comparison of β-diversity at different caries stages

**Fig 2:**
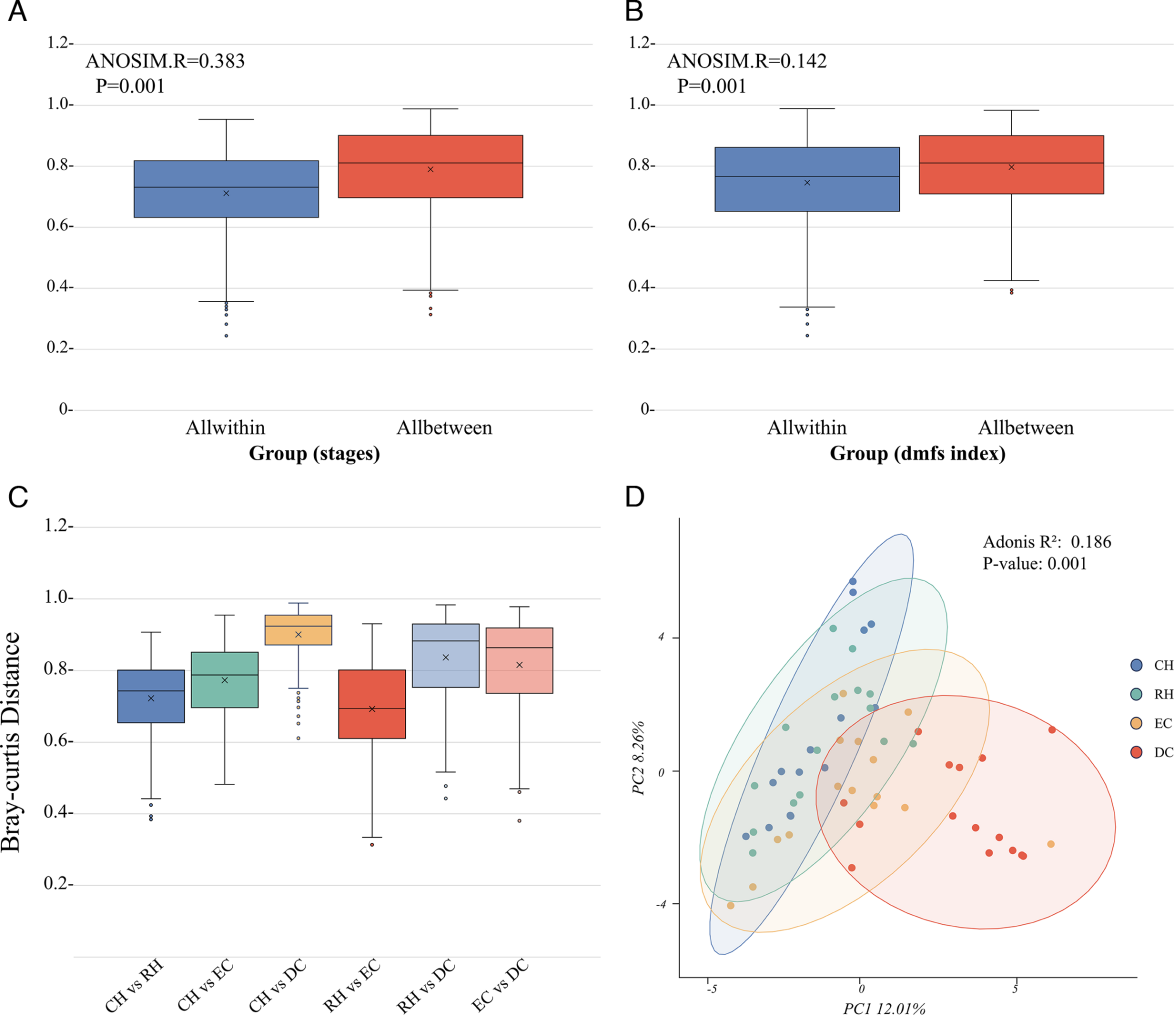
Changes in the β-diversity (Bray-Curtis distance) of the bacterial community. (**A**) Comparison of the microbiota composition in β-diversity between within-group and between-group according to different stages. (**B**) Comparison of the microbiota composition in β-diversity between within-group and between-group according to dmfs. (**C**) Comparison of all between-group distances according to different stages. (**D**) PCA generated using Bray-Curtis distance indicates distinct clustering of samples from each group.

We calculated within-group and between-group Bray-Curtis distances to assess the impact of different factors, including “caries stages” and “dmfs,” on the microbiota community structure. The boxplot showed that the above two factors both significantly influenced microbial community structure (Fig. 2A and B). Moreover, the greatest distance was measured in the CH group to the DC group, implying the most tremendous microbial structure difference between “confident health” and “deepest caries lesions.” The distance between the RH/EC group and the DC group was followed, indicating the distinct microbial community structure in the deepest lesions of the DC group. The smallest distance was observed between the RH and EC groups, demonstrating the resemblance of microbial structure between enamel caries and non-cavitated tooth surfaces, even in the absence of clinically visible lesions. In the PCA plot, the DC group exhibited a relatively dispersed distance from the other groups (Fig. 2D). Other samples from CH, RH to EC progressively aligned sequentially along with the dental caries advancement, indicating gradual changes in microbial structure in the caries progression process (Fig. 2D).

### The comparison of species composition at different caries stages

**Fig 3:**
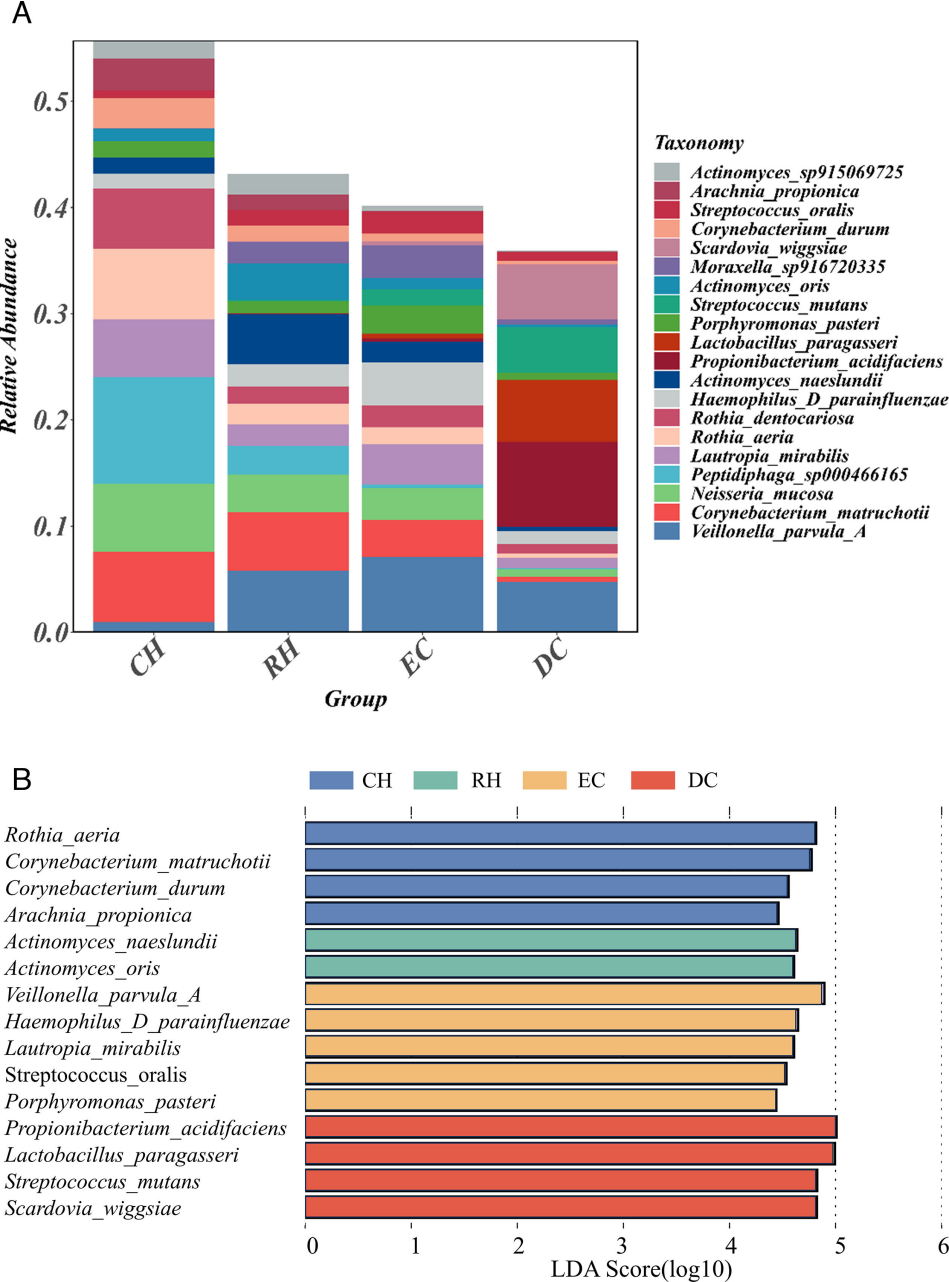
Analysis of the composition and differences of dominant bacteria in different groups. (**A**) Bacterial composition (relative abundance) of different groups at the species level. Each color represents a species. (**B**) Dominant species with significant differences in different groups were detected by LDA effect size (LEfSe) analysis (LDA > 4.0, *P* < 0.05). Each color represents a group.

To further characterize the dental plaque in the development stage of caries, we compared the relative abundance of dental plaque at the genus and species levels in four stages. First, in the bacterial kingdom, we analyzed the overall structure of the oral microflora in each group. Interestingly, the proportions of dominant bacteria (relative abundance ≥1%) in each group exhibited significant differences. (Fig. 3A). We performed a linear discriminant analysis effect size (LEfSe) to identify differences in relative abundant species among groups (Fig. 3B). The results showed that high LDA scores were observed for *Propionibacterium_acidifaciens*, *Lactobacillus_paragasseri*, *Streptococcus_mutans*, and *Scardovia_wiggsiae* in the DC group, for *Actinomyces naeslundii* and *Actinomyces oris* in the RH group, and *Rothia_aeria*, *Corynebacterium_matruchotii*, *Corynebacterium_durum*, and *Arachnia_propionica* in the CH group.

**Fig 4:**
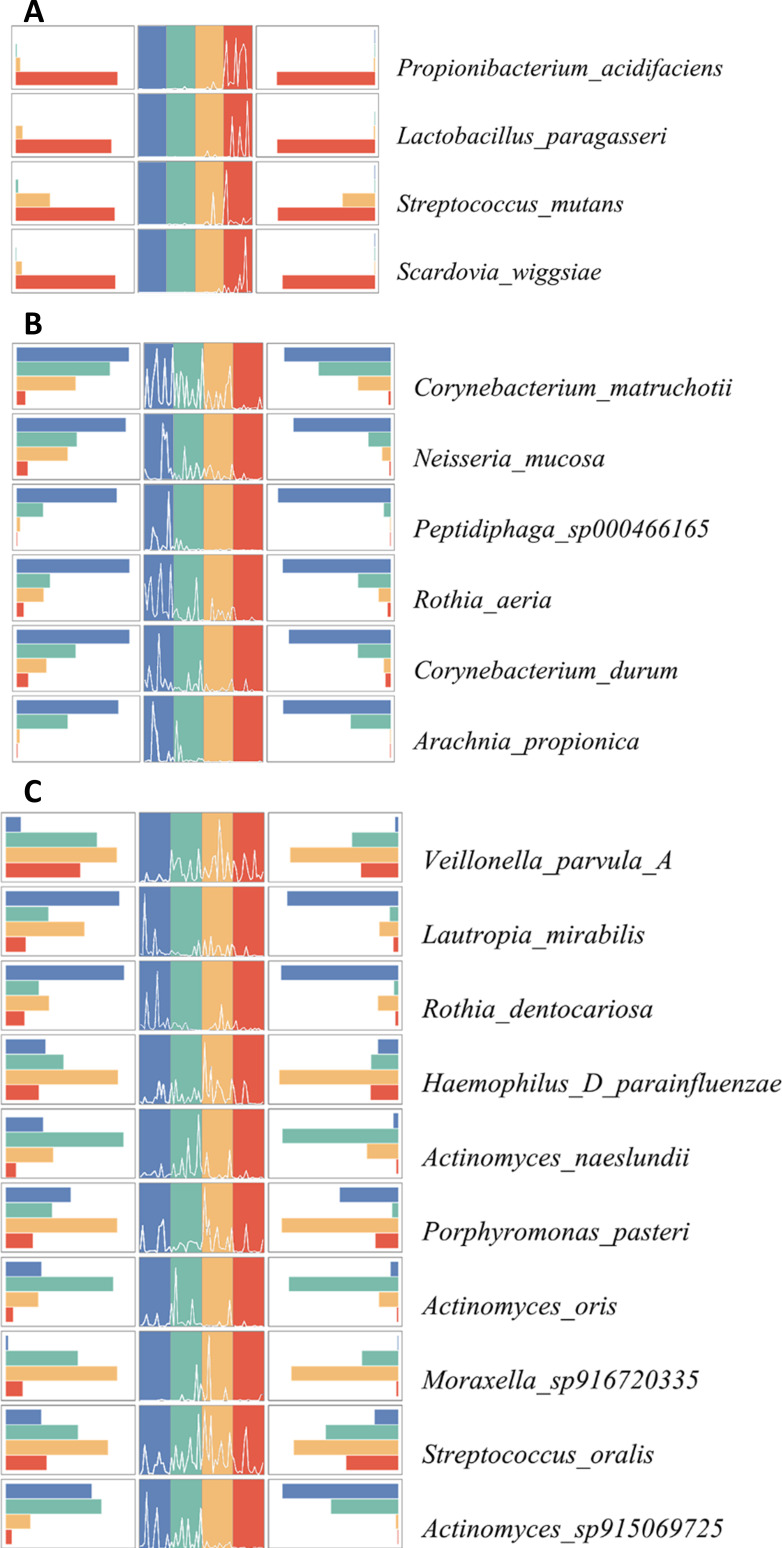
Trends in the relative abundance of dominant bacteria at different caries stages. (**A**) Increasing trend charts of dominant species during the progression of dental caries. (**B**) Decreasing trend charts of dominant species during the progression of dental caries. (**C**) Unclear trend charts of dominant species during the progression of dental caries. The left plot is a histogram showing the change in average relative abundance, the middle plot is a line chart showing the trend change in each sample, and the right plot is a histogram showing the variance of the relative abundance in each group. Each color represents a group.

We found the upward trends of the following four dominant species along with the caries progression, including *P. acidifaciens*, *S. mutans*, *L. paragasseri*, and *S. wiggsiae* (Fig. 4A). By contrast, some species, such as *C. matruchotii*, *N. mucosa*, *Peptidiphaga_sp000466165*, *R. aeri*, *C. durum*, and *A. propionica*, showed a decreasing trend during caries progression (Fig. 4B). In addition, certain dominant species showed unclear trend changes (Fig. 4C).

At the same time, 20 dominant genera were found at the genus level, including *Neisseria*, *Streptococcus*, *Actinomyces*, *Prevotella*, *Veillonella*, and so on. The overall structure of the oral microbiota for each group is shown in Fig.S2A. The system of dominant genera in the DC group changed significantly, and the composition of the CH, RH, and EC groups is similar. LEfSe also identified several differentially abundant taxa at the genus level (Fig. S2B). These results showed that high LDA scores were observed for *Prevotella*, *Propionibacterium*, *Lactobacillus*, *Scardovia*, and *Allprevotella* in the DC group. Besides, similar trend changes were also observed at the genus level. The trends of dominant genera showed five increased genera (including *Prevotella*, *Propionibacterium*, *Lactobacillus*, *Scardovia*, and *Allprevotella*) and six decreased genera (including *Neisseria*, *Rothia*, *Corynebacterium*, *Lautropia*, *Peptidiphaga*, and *Arachnia*) (Fig. S3A through C).

The 2bRAD-M technique can also provide information on the abundance of archaea and fungi, in addition to bacteria. The relative abundance of archaea is nearly zero, with only a few samples occasionally containing archaea (Fig. S4A). By contrast, fungi are detected in a larger number of samples, with *Malassezia restricta* and *Candida albicans* being the most dominant species. Compared with the CH group, *Candida albicans* mainly existed in the RH, EC*,* and DC groups (Fig. S4B).

### The diagnostic model based on deep learning shows a more stable diagnostic performance

The research results above indicated changes in the microbial community structure as the transition occurs from totally healthy to dentin caries. However, the RH group and EC group were very similar in microbial structure, and it was difficult to distinguish them. Therefore, it was crucial to develop a caries diagnostic model to pinpoint different stages, especially the RH, for disease prevention, diagnosis, and treatment.

**Fig 5:**
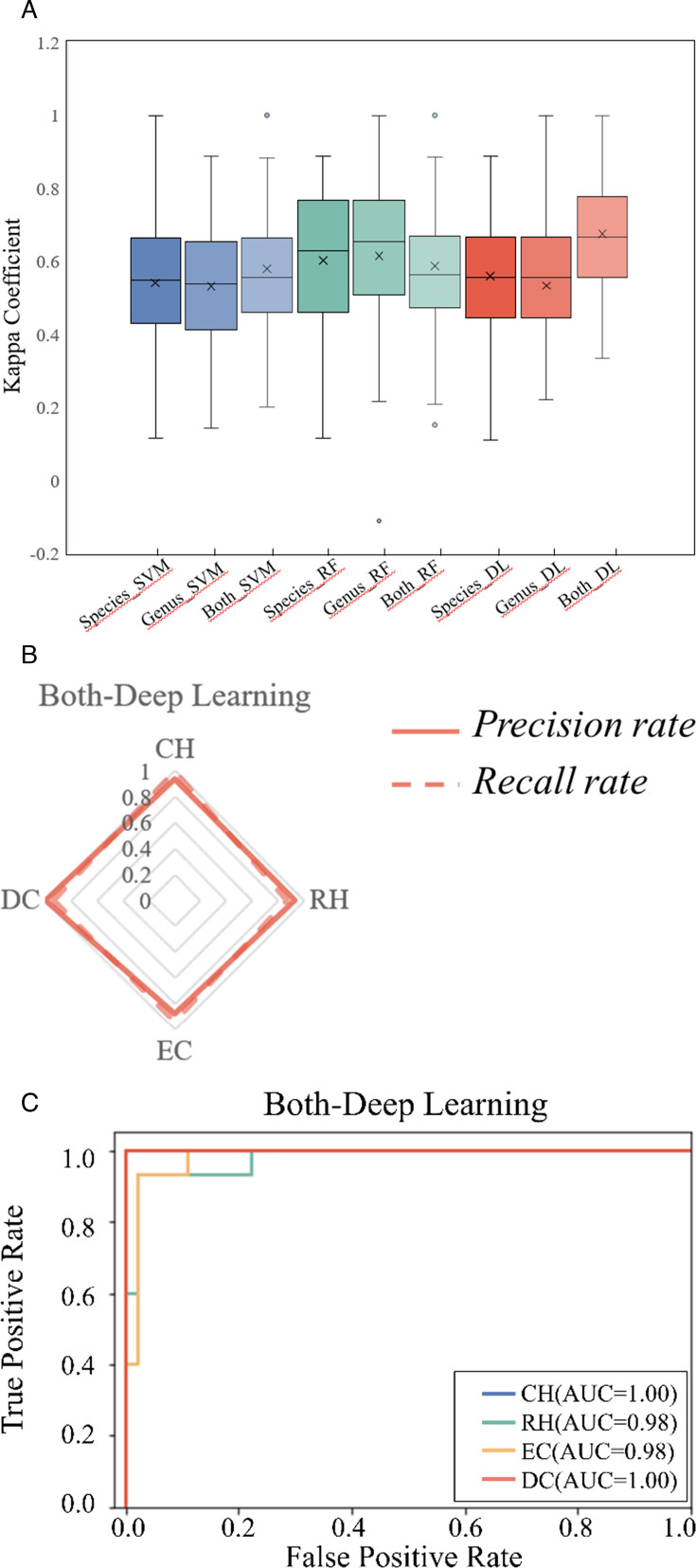
Transformation among four stages and evaluation of diagnostic models for different caries stages. (**A**) Comparison of deep learning-based and model-based (SVM and RF) methods for performance via Kappa coefficients. (**B**) Recall and precision of deep learning models. Each vertex of the pentagon represents a recall/precision value on specific stages. (**C**) Receiver operating characteristics (ROCs) of deep learning based on different caries stages diagnosis.

To provide a new biological basis for different caries stages diagnosis and treatment, we explored classical machine learning methods such as support vector machines (SVM), random forest (RF), and deep learning based on neural networks (DL). We incorporated microbial data at the genus and species levels and innovatively combined both levels to construct and evaluate the diagnostic models. To test the accuracy of these models, we performed a fivefold cross-validation on the data set and repeated it 100 times. Subsequently, we compared the overall performance of these diagnostic models using evaluation metrics such as the kappa coefficient (k-value), recall rate, precision rate, and ROC. The results showed that the deep learning model combining genus and species (Both_DL) information outperformed the other models. The k-value, recall rate, precision rate, and ROC of the Both_DL model were significantly higher than those of the other models (*P* < 0.01) (Fig. 5A through C; Fig. S5 and S6). These findings indicated that the diagnostic model constructed by combining genus and species information using deep learning methods based on neural networks offers superior and more stable diagnostic performance.

**Fig 6:**
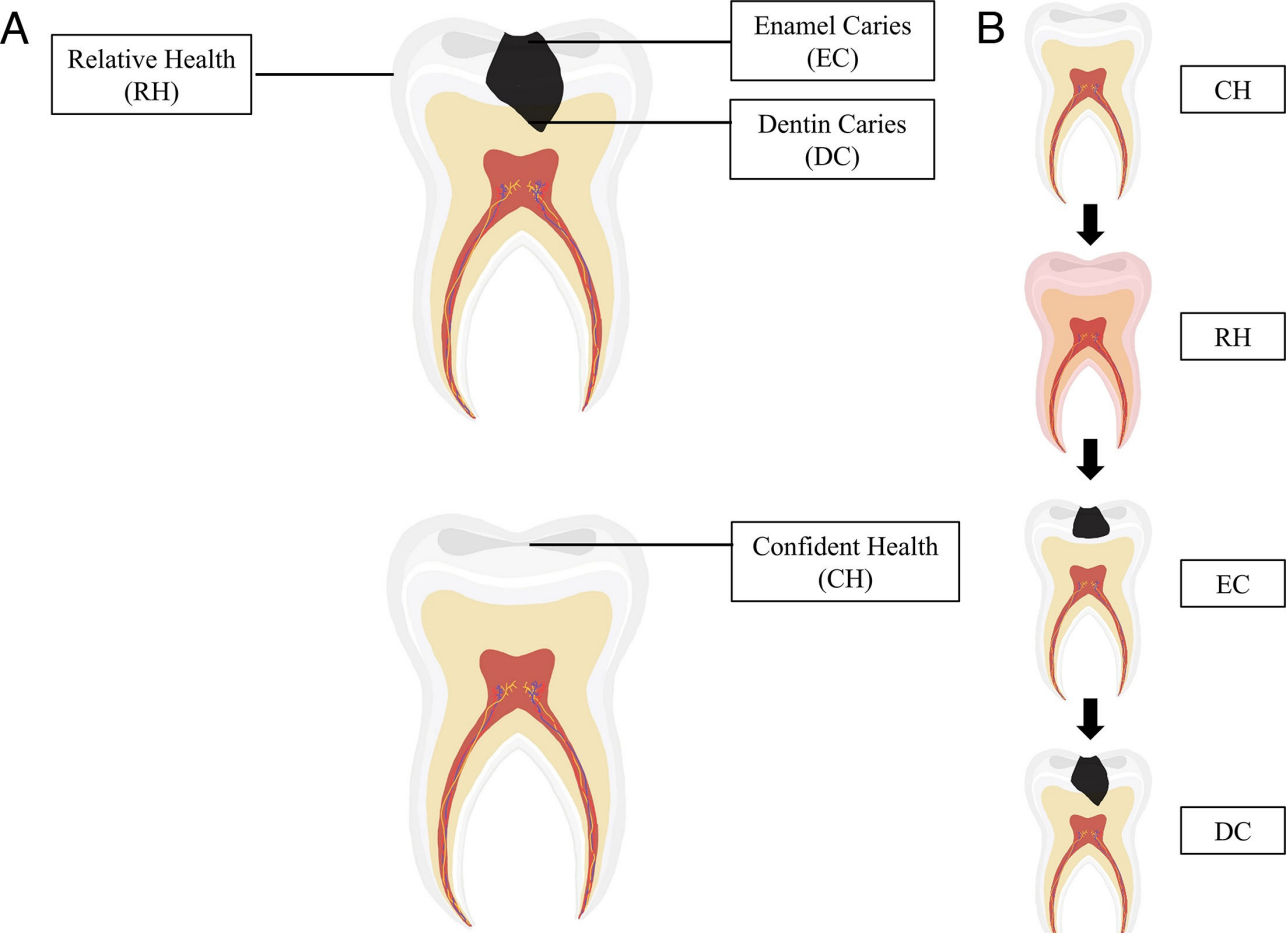
Schematic diagram of sample collection. (**A**) Sampling schematic diagram of deep caries and confident health. The RH group was collected from the non-cavitated surface on the caries-active, and EC and DC groups were collected from the enamel and dentin layers. (**B**) Progression of caries from caries-free to dentin caries.

## DISCUSSION

The emergence of high-throughput sequencing technologies has provided a broader platform for the study of oral microbial communities along with dental disease. In our study, we adopted a novel metagenomic sequencing technique, 2bRAD-M, to analyze the microbial changes in different caries status samples to gain deeper insights of information with caries at the species level with high resolution. This study aimed to investigate the microbial differences among completely healthy individuals and those with caries samples at various stages from caries-active individuals. Although the completely healthy samples (CH) were exclusively obtained from healthy individuals, we found no significant differences in species diversity among individuals (Fig. S1). Moreover, the principal component analysis (PCA) results demonstrated that the microbial community structure in the dentin caries (DC) is notably distinct from all other samples. Therefore, the individual heterogeneity may be undeniable in this study; however, the variation among samples from different caries stages significantly surpasses the differences observed among individuals. That is why we could develop the diagnostic models for various stages of caries progression based on the divergences of bacterial lineages.

First, our research highlighted that the highest species diversity was found in those extremely healthy states (CH). In addition, our analysis of the Bray-Curtis distance revealed a relatively large distance between dentin caries (DC) and all other groups. These findings were consistent with the ecological plaque hypothesis (24). In a totally healthy state (CH), teeth provide a mild and suitable environment that facilitates the growth and reproduction of various species. However, frequent intake of sugars and carbohydrates contributes to the progression of completely healthy teeth to carious states. Dentin caries (DC), as the deepest layer in this progression, exists in a relatively closed environment characterized by a lack of oxygen and salivary buffering, creating a more challenging living condition (25). In this stringent ecological niche, only specific acidic-enduring taxa can survive, resulting in a significant change to the microbial structure in the DC group. And these results corroborated those findings from previous research (26, 27). Interestingly, we noted the distance between the RH and EC groups was the smallest, which emphasized the alterations in microbial community structure that occurred in the process of the “pre-caries” period ahead of clinical identification. It highlights the importance of early intervention and prevention management of caries to those high-risk individuals, even if no clinical caries can be pinpointed.

Second, LEfSe analysis identified species with significant differences in abundance in each group. *Streptococcus mutans*, *Propionibacterium acidifaciens*, *Lactobacillus paragasseri,* and *Scardovia wiggsiae* showed a significantly increased trend in the DC group. *S. mutans* was widely recognized as a critical pathogen in dental caries in driving the disease. *Propionibacterium* was reported to have the ability to tightly bind to collagen proteins in dentin, and its acid resistance characteristics contribute to the progression of dentin caries (28, 29). *L. paragasseri* is the major constitutive member of *Lactobacilli* present in dentin caries samples, suggesting its role in colonization and proliferation in decaying dentin (30). *S. wiggsiae* was reported to possess a unique metabolic pathway, the F6PPK shunt, which might contribute to its high ecological competitiveness in acidic environments like caries lesions (31). Moreover, the LEfSe analysis at the genus level revealed a significant differential abundance of *Prevotella* in dentin caries. The above result is in accord with our previous cross-sectional and longitudinal studies of finding *Prevotella* as caries-associated and its critical role in the construction of the caries predicting models (32–36). In 2013, Simón-Soro et al. conducted whole-genome sequencing on plaque samples and discovered that the overexpression of collagenase genes in dentin caries was primarily attributed to the increased abundance of *Prevotella*. The above results all suggested that *Prevotella* may play a role in the development of dental caries through its ability to dissolve collagen proteins and its specific sugar metabolism characteristics (17).

Contrarily, four species, including *Rothia_aeria*, *Corynebacterium_matruchotii*, *Corynebacterium_durum*, and *Arachnia_propionica,* were significantly more abundant in the *CH* group. *Corynebacterium* has been extensively studied and is known to play a significant role in maintaining oral health and ecological balance (37). *C. durum*, both directly and via its extracellular membrane vesicles, disrupts interkingdom assemblages between *Candida albicans* and *Streptococcus mutans* (38). *C. matruchotii*, in particular, can utilize lactate produced by other cohabiting plaque microbes, potentially raising the pH in the oral cavity (39, 40). *R. aeria* is a nitrate-reducing microorganism that plays a role in enhancing resilience against salivary acidification. This attribute aids in the prevention of dental caries formation and the preservation of oral environment homeostasis (41, 42). Moreover, in the RH group, a greater proportion of *Actinomyces* was observed, associated with the ecological balance of biofilm and the promotion of dental plaque formation (43, 44). However, some previous studies suggested that *Actinomyces* may exhibit pathogenicity when exposed to acidic environments for a prolonged period (45, 46). Further exploration is still needed to understand the mechanisms by which these species could participate in the critical change of microbial members driving the caries occurrence.

In addition to the promotion or inhibition effects of bacteria on the development of dental caries, some fungi, such as *Candida albicans*, also play a certain role in the progression of dental caries. The fungi could adhere to the hydroxyapatite (HAP) substrates and damage the crystal structure by releasing calcium ions. Furthermore, some studies also indicated that *C. albicans* and *S. mutans* interacted synergistically in driving the progression of caries. Compared to single-species biofilms, dual-species biofilms composed of *S. mutans* and *C. albicans* could enhance the overall virulence of cariogenic biofilms (47).

Finally, this study employed neural network-based deep learning techniques to establish a precise and dependable diagnostic model for dental caries. In contrast to conventional machine learning algorithms (48–50), we incorporated all microbial data from the study in the model training to balance the interaction among microbial communities. As a result, our model achieved an area under the receiver operating characteristic curve of 100%, 98%, 98%, and 100% for confident health (CH), relative health (RH), enamel caries (EC), and dentin caries (DC), respectively. In the field of dentistry related to deep learning, the majority of previous studies utilized a combination of deep learning and X-ray images to detect dental caries, with the average AUC of 84.9% for diagnosing dental caries (51–55). However, our research showed the integration of deep learning with high-throughput sequencing data, constructing models suitable for diagnosing different dental caries stages and offering promising prospects in the field. This advancement has the potential to promote the development of rapid chairside testing and self-testing for home care, improving the overall management and prevention of dental caries.

We have to admit the limitation of not having a very large sample size in the present study. Most of the previous similar research focused on the plaque microbiomes from those categorized subjects based on the caries severity, with complex and different individual genetic background factors involved (19, 20, 27, 56). In our study, despite the limited volunteer number of 30, the origin of CH is 15 volunteers, and the other 15 volunteers are the source of the 45 plaque samples, including RH, EC*,* and DC. It is noteworthy that the PERMANOVA result showed “different stages of caries” is the dominant grouping factor with the highest R-squared, followed by the “dmfs index.” It indicated that the microbial divergence among the different disease stages has outperformed the genetic background of those volunteers in this sample size. Therefore, this study represents a microscale investigation into the microbial disparity across various stages of dental caries progression, facilitated by microbial community profiling with species-level taxonomic resolution. Future research will increase the sample size for more comprehensive findings, aiming for the development of novel strategies for the diagnosis and prevention of caries.

### Conclusion

In this study, we conducted metagenomic sequencing analysis of 60 samples at different stages of caries development. Notably, this study marks the first instance of 2bRAD-M sequencing in the analysis of the oral microbiome. Our findings revealed that the confident healthy dental plaque exhibited the greatest diversity of microbial species. Furthermore, our findings highlighted the importance of diagnosing and preventing issues in sub-health to maintain oral health. Once the dentin layer became affected, significant shifts occurred in microbial composition and community structure. We also found notable shifts in dominant species abundance. In addition, we successfully applied the combination of oral microbiome data and deep learning techniques to diagnose different caries stages. This breakthrough holds promise for the development and implementation of rapid chairside tests in clinical practice or for self-monitoring in at-home oral care.

## MATERIALS AND METHODS

### Study design and recruitment

The inclusion criteria were sound and caries-active deciduous molars. The exclusion criteria were teeth with pulp infection, antibiotics in the previous 3 months, and currently undergoing dental treatment or a topical oral antimicrobial agent. The sample size calculation was performed with reference to previous similar studies (16, 19, 57). In total, we recruited 15 samples in each group, and the total sample size needed was 60 samples. Validation through rarefaction curves confirmed that 15 samples per group fulfilled the minimum sample size required for this experiment (Fig. S7A through D). Thirty children aged 3–7 years old were selected through convenience sampling.

A calibrated pediatric dentist collected 60 dental plaque samples (Fig. 6): 15 confident health samples (buccal surface on caries-free teeth, defined as confident health [CH]), 15 relative health samples (non-cavitated buccal surface on caries-active, defined as relative health [RH]), 15 enamel caries samples (enamel layers on caries-active [EC]), and 15 dentin caries samples (dentin layers on caries-active [DC]). The CH group samples were collected from the buccal surface in deciduous molars of healthy individuals. The samples from the RH group surface were collected from the spatially paired “healthy” sites, the non-cavitated buccal surfaces of caries-active deciduous molars. We ensured that bacterial biofilm from the non-cavitated buccal was taken from the same patient with carious lesions (enamel/dentin). The procedure was confirmed through clinical and imaging evaluations by professional dentists. We used sterile swabs to collect samples from healthy teeth and buccal surfaces, while sterile dental excavators were employed to obtain samples from enamel and dentin lesions. The collected samples were immersed in a sterile 1.5 mL Eppendorf tube with 1 mL of UV-irradiated 95% alcohol to avoid DNA contamination. The samples were transported on ice to the laboratory and stored at −80°C until used.

### Library construction and sequencing

The 2bRAD-M library preparation followed the original protocol developed by Wang et al. (58) with minor modifications. DNA (1 pg–200 ng) was digested with 4 U of the enzyme BcgI (NEB) for 3 h at 37°C. Subsequently, the adaptors were ligated to the DNA fragments. The ligation reaction was performed by combining 5 µL of digested DNA with 10 µL of a ligation master mix containing 0.2 µM each of two adaptors and 800 U T4 DNA ligase (NEB). Ligation was carried out at 4°C for 12 h. Then, ligation products were amplified, and PCR products were subjected to 8% polyacrylamide gel. Bands of approximately 100 bp were excised from the polyacrylamide gel, and the DNA was eluted from the gel in nuclease-free water for 12 h at 4°C. Sample-specific barcodes were introduced by PCR with platform-specific barcode-bearing primers. Each 20 µL PCR contained 25 ng of gel-extracted PCR product, 0.2 µM of each primer, 0.3 mM dNTP, 1× Phusion HF buffer, and 0.4 U Phusion high-fidelity DNA polymerase (NEB). PCR products were purified using a QIAquick PCR purification kit (Qiagen) and then subjected to sequencing using the Illumina Nova PE150 platform. 2bRAD-M was carried out at the Qingdao OE Biotech Co., Ltd. (Qingdao, China).

### Data quality control of raw data

The raw data were preprocessed to construct a species-level Unique Tag Database (Classification was carried out by utilizing the unique 2bRAD tag database that includes bcgI-derived tags of taxonomic species from 173,165 microbial genomes, referred to as 2b-Tag-DB). All sequenced 2bRAD tags after quality control were mapped (using a built-in Perl script) against the 2bRAD marker reference database. To control the false positive in species identification, a G score was derived for each species identified within a sample. False-positive filtering was performed based on the G score threshold (set at 10) to screen candidate microorganisms. Finally, the unique tag database of each sample’s candidate microorganism species level was constructed again; the clean reads were compared with 2b-Tag-DB for qualitative analysis, and the relative abundance of each microbial species was estimated. The formula is as follows: relative abundance _species *i*_ = $\frac{S_{i}/T_{i}}{\Sigma_{\mathrm{i}=1}^{n}S_{i}/T_{i}}$ (where *S* represents the total number of read lengths of all 2bRAD tags mapped to species *i* in the sample and *T* represents the total number of 2bRAD tags of species *i* in the database).

### Diversity analyses and LEfSe

To analyze the diversity of bacterial microbiota among the groups, α- and β-diversity analyses were performed using the R script of the Parallel-Meta software package. Analysis indices were the Chao1 index for α*-*diversity and the Bray-Curtis distance for β*-*diversity. Effect size (ES) is a numerical value that quantifies the strength of a phenomenon, which is used to make up for the misjudgment of the results caused by over-reliance on the *P*-value (*P*). In this study, we characterized the ES according to Cohen’s *d* to evaluate the impact strength of different influencing factors, including the dmfs index (the number of decayed, missing, and filled tooth surfaces in deciduous dentition), age, and tooth site. LEfSe analysis was performed to determine the characteristic bacteria for each group. In the results of LEfSe, particularly high linear discriminant analysis (LDA) scores (>4.0) were extracted and used as candidates for caries-associated species.

### Construction of diagnostic models

#### Deep learning model training process and comparison

In this study, the data set was divided into D1 and D2 with a ratio of 8:2, and D1 was used to train the deep learning model. Currently, there is no standard method to determine the parameters of the neural network model (such as the size of the fully connected layer and the number of layers). The usual practice is to set a parameter range and find suitable parameters within it to optimize the performance of the neural network model. In this study, to ensure accurate feature extraction from the input data, the input layer was set within the range of 32 to 128. Since the data set in this study consists of only 60 samples, a deep network is not needed. Therefore, the hidden layers were set within the range of 2–5. To avoid overfitting, the dropout technique was introduced, which randomly disables some input neurons. The dropout rate is controlled by the hyperparameter DROPOUTS, and its range is set from 0.5 to 0.9. By doing this, we encourage the network not to overly rely on specific neurons during the training process, thereby enhancing the model’s generalization ability. Since this study is a four-classification task, the neural network ends with an output layer consisting of four neurons. Softmax activation function was used to adapt to the multi-classification problem and map the original output of the network to a probability distribution, with each class represented as a dimension of the output vector. Our model used the Adam optimizer, which adaptively adjusts the learning rate to effectively optimize the weights of the model. The loss function of the model was set as categorical cross-entropy, which is widely used in multi-classification problems and effectively measures the difference between model predictions and actual labels. GridSearch was then used to train the neural network within the parameter range we set, and the best parameters were selected. The code was implemented in Python (3.8) using the Keras and sklearn packages. In addition, the performance of two commonly used classifiers, support vector machine (SVM) and random forest (RF) models, was evaluated and compared with the neural network model using the sklearn package.

#### Comparison of the three models at the species level

We used RF, SVM, and deep learning (DL) models to identify species associated with dental caries-related biomarkers. For the DL model, we used mutual information to calculate the feature weights of all species in the D1 database at the species level to determine their impact on the classification output. Based on these scores, we ranked all species in the D1 samples. We then selected the top 100 species with the greatest impact as biomarkers related to changes in oral dental caries status. Next, we trained the model again using the data of the top k-ranked species (k ranging from 5 to 100) to find the optimal k value that maximizes the classification performance of the model. Finally, we tested the model using the D2 data set and compared the predictive performance of using all species data and using the biomarker species data. For the RF model, we used the Random Forest Classifier function from the sklearn library, and the rest of the process was the same as the DL model. For the SVM model, we used the SVC function from the sklearn.svm library, and the rest of the process was the same as the DL model. Similar to the comparison at the species level, here we used the biological information of the D1 database samples at the genus level.

#### Judging the status of dental caries using the genus and species levels

Combining the genus and species levels can provide more comprehensive and integrated information, better reflecting the overall composition of microbial samples. This can reduce information loss and generate more accurate biological explanations. By integrating information from different levels, it can reveal the complex interactions within microbial communities, thereby providing deeper insights for bioinformatics and ecological research. We selected the top 100 markers with the highest scores for genus and species in the D1 sample, combined them as input data to train random forests (RF), support vector machines (SVM), and DL models. Finally, we used the D2 data set for testing to evaluate the predictive performance of the models.

### Statistical analysis

Statistical analysis was performed using the Wilcoxon test for pairwise comparison between groups; the Kruskal-Wallis test was used for comparison between multiple groups, and the Adonis analysis was used for the differences in microbial community structure between groups. Statistical significance was set at *P* < 0.05.

To study the performance of random forests (RF), support vector machines (SVM), and DL models in the classification task of dental caries status, we used accuracy, precision, recall, and ROC curve. Accuracy measure reflects the overall quality of the classifier. Precision measure focuses on the accuracy of the classifier’s predictions for the current status of dental caries. Recall measure answers how well the classifier can identify the corresponding samples of dental caries. Furthermore, we also conducted ROC curve and AUC measurement. The ROC curve shows the relationship between the true-positive rate (the proportion of correctly predicted positive samples to the total predicted positive samples) and the false-positive rate (the proportion of incorrectly predicted positive samples to the total predicted negative samples). AUC measurement ranges from 0.5 to 1 (the higher the value, the better the performance) and is derived from the ROC curve. It summarizes the false-positive rate and true-positive rate.

## Data Availability

All data files are available from the NCBI database (PRJNA1268289).

## SUPPLEMENTAL MATERIAL

Supplemental material msystems.00044-25-s0001.docx Table S1 and Fig. S1 to S7.

ASM does not own the copyrights to Supplemental Material that may be linked to, or accessed through, an article. The authors have granted ASM a non-exclusive, world-wide license to publish the Supplemental Material files. Please contact the corresponding author directly for reuse.
