# Supplementary material for: Metagenomic research on the structural difference of plaque microbiome from different caries stages and the construction of a caries diagnostic model
Source: mSystems. 2025 Sep 10;10(10):e00044-25. doi: 10.1128/msystems.00044-25 (PMC12542677; doi:10.1128/msystems.00044-25)
Supplement: Supplemental material — Table S1 and Fig. S1 to S7. [file msystems.00044-25-s0001.docx]

| **Group** | **R²** | ***P*** |
| --- | --- | --- |
| Different stages | 0.186 | 0.001^***^ |
| Age | 0.041 | 0.007^**^ |
| Tooth | 0.025 | 0.079 |
| dmfs index | 0.071 | 0.001^***^ |

**Table.S1 Permutational multivariate analysis of variance (PERMANOVA) using Bray-Curtis distanc (*****P*＜0.01， ****P*＜0.001)


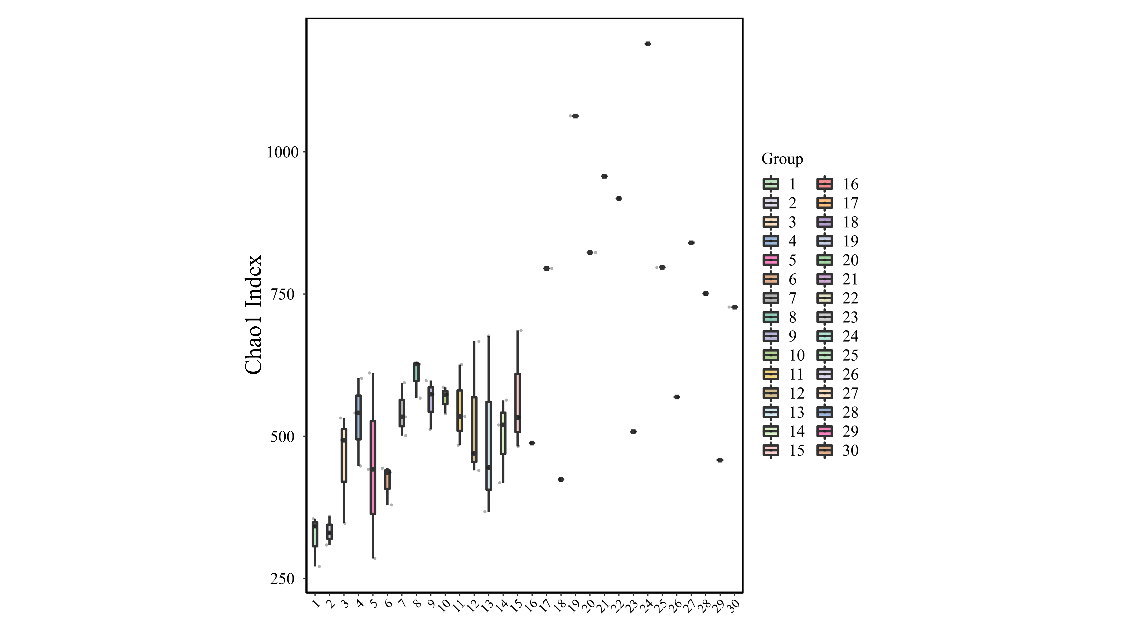


**
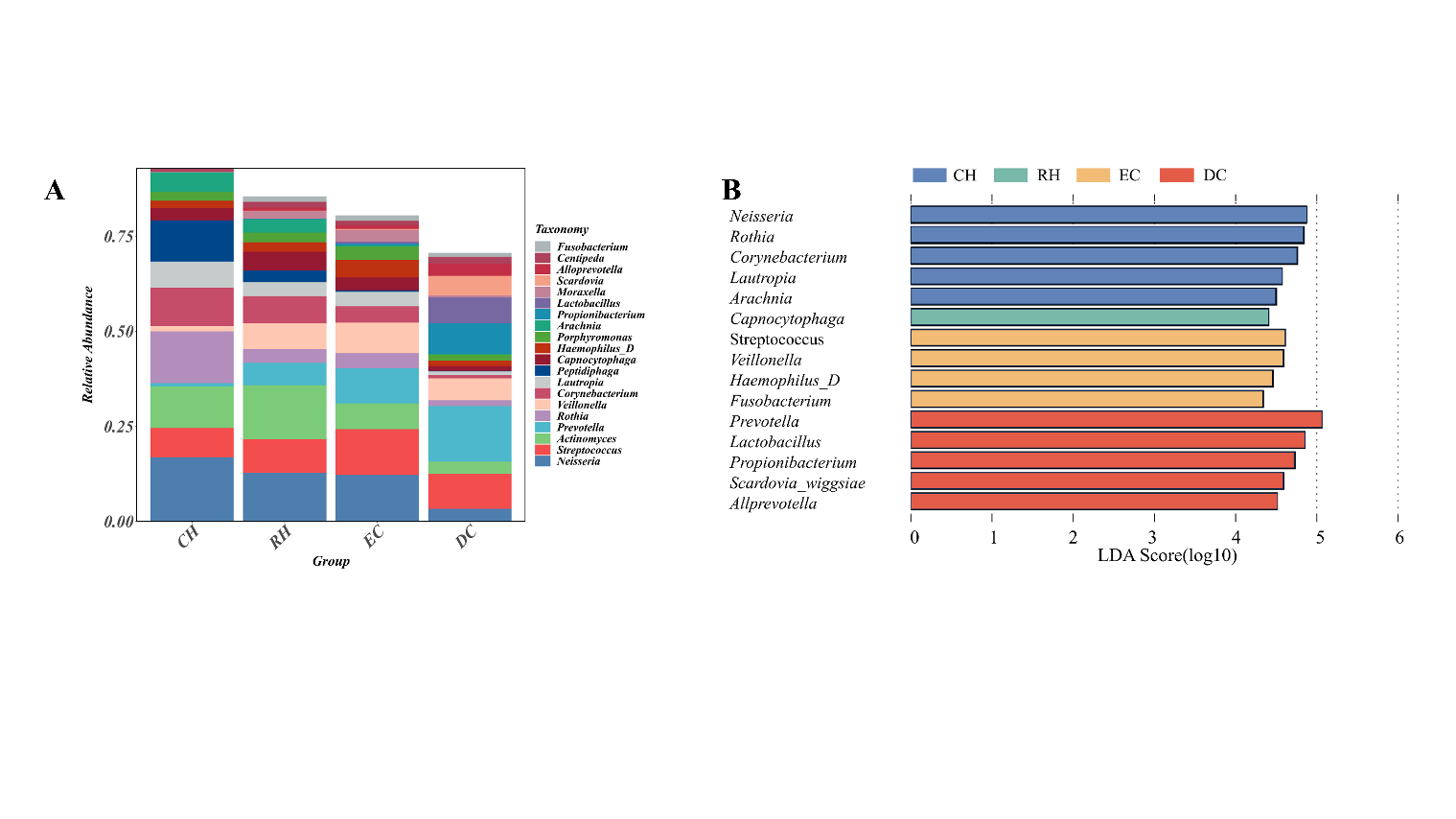
Figure. S1** **Boxplot of species diversity according to different individuals *(P*＞0.05)**

**Figure. S2 Dominant genera composition and trend changes at different stages**

(A) Bacterial composition (relative abundance) of different groups at the genus level. Each color represents a species. (B) Dominant genus with significant differences in different groups were detected by LDA effect size (LEfSe) analysis (LDA > 4.0, P< 0.05). Each color represents a group.

**
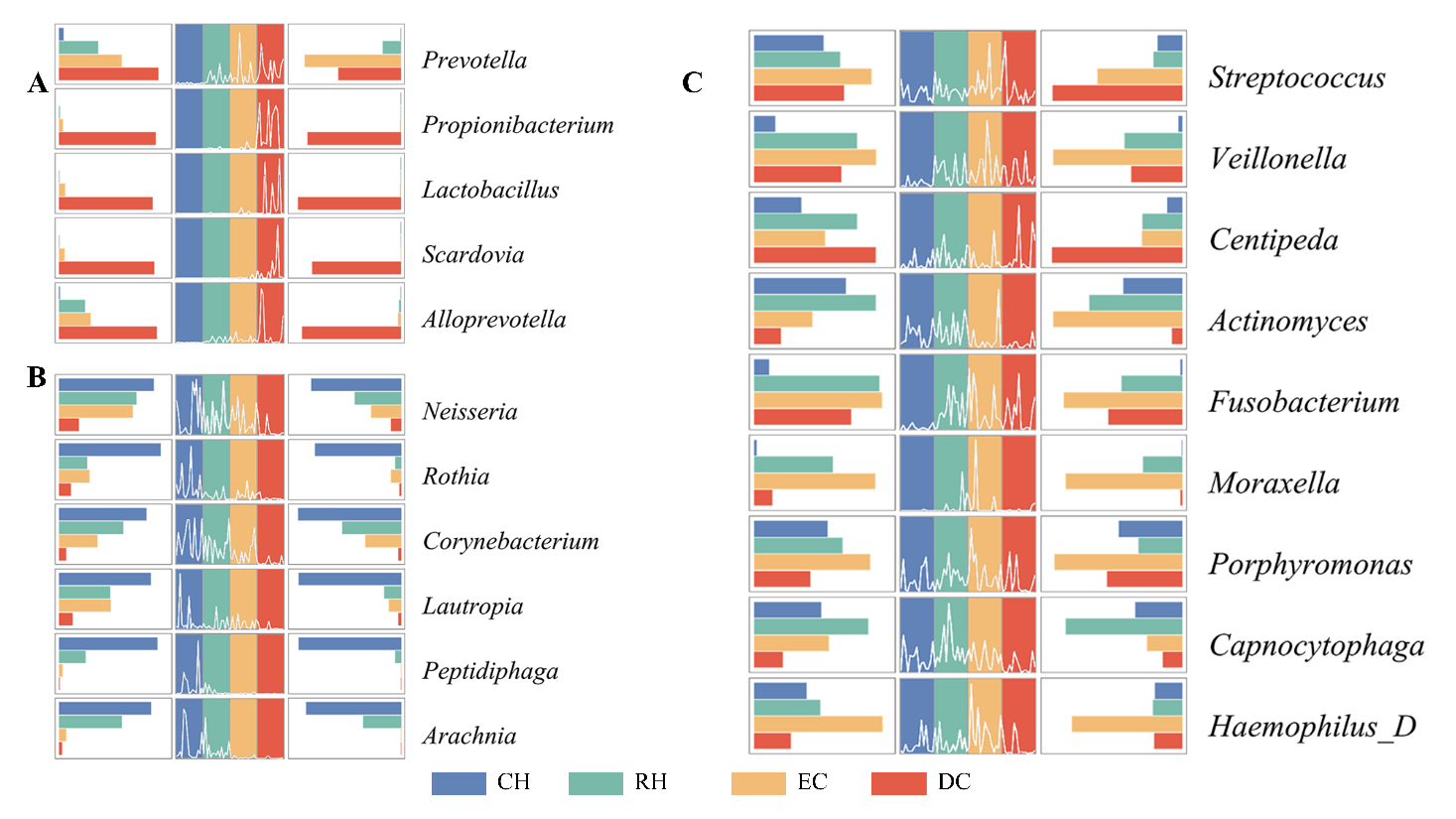
Figure.S3 Trends in the relative abundance of dominant genera at different caries stages**

(A) Increasing trend charts of dominant genera during the progression of dental caries. (B) Decreasing trend charts of dominant genera during the progression of dental caries. (C) Unclear trend charts of dominant genera during the progression of dental caries.


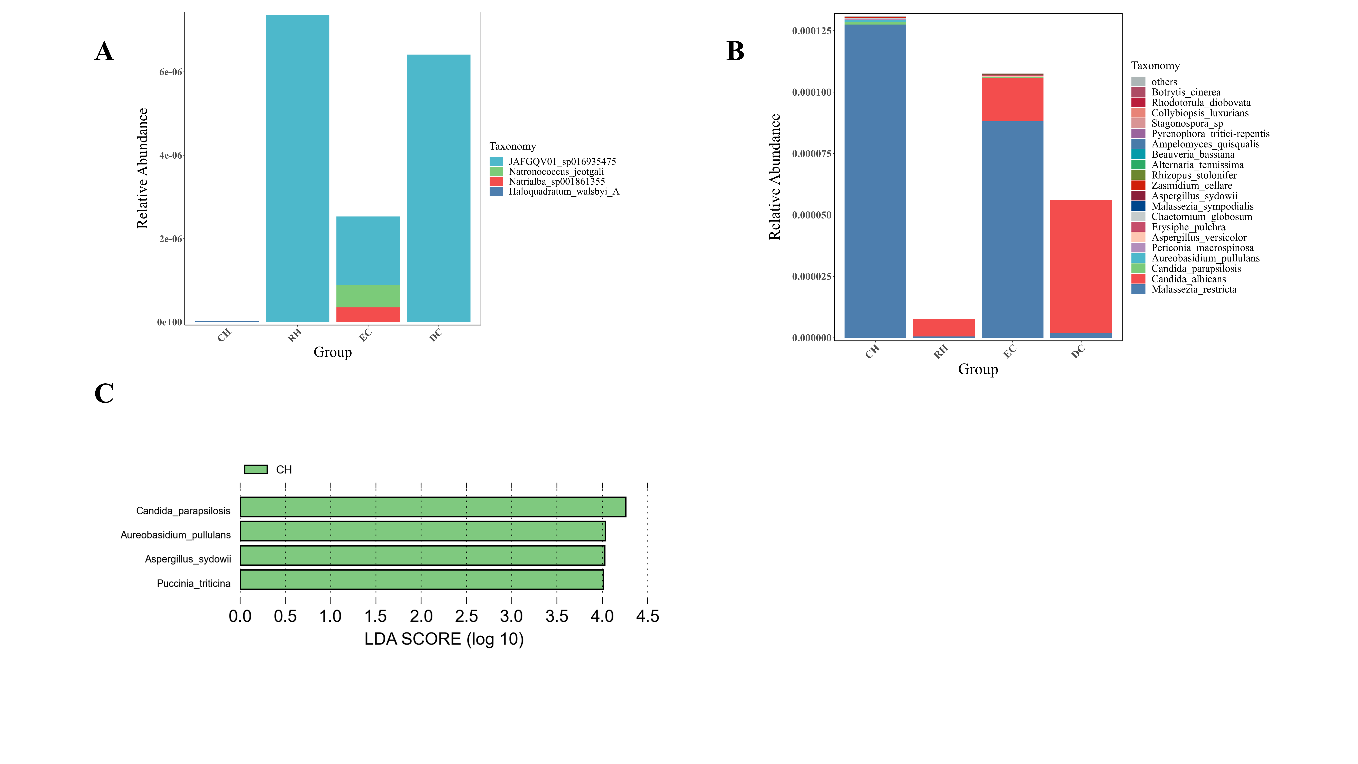
The left plot is a histogram showing the change in average relative abundance, the middle plot is a line chart showing the trend change in each sample, and the right plot is a histogram showing the variance of the relative abundance in each group. Each color represents a group.

**Figure. S4 Dominant species composition in the archaea and fungi kingdom**

(A) Species composition (relative abundance) of different groups at the archaea kingdom. Each color represents a species. (B) Species composition (relative abundance) of different groups at the fungi kingdom. Each color represents a species.


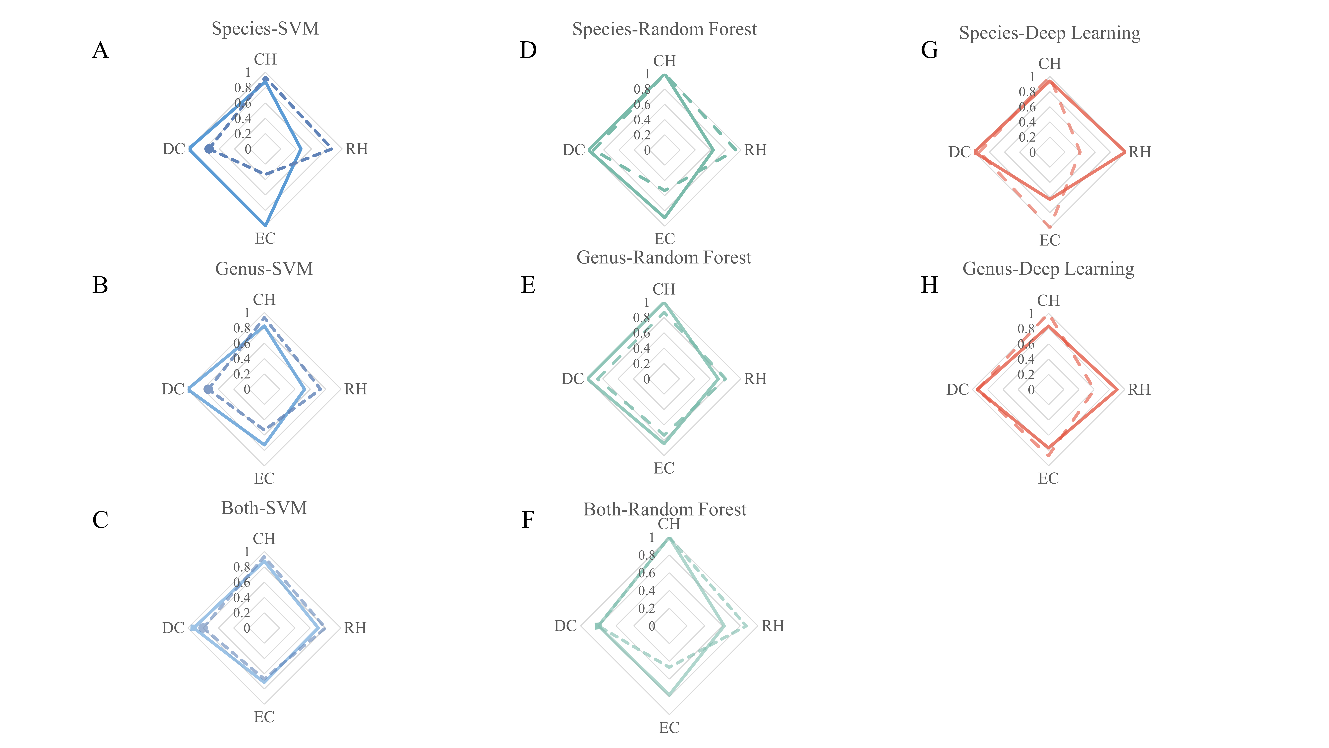


**Figure.S5 Comparison of precision rate and recall rate of other eight models**

The solid line represents the precision, and the dashed line the recall. The larger the precision/recall value, the better the performance. (A) SVM combine with species data. (B) SVM combine with genus data. (C) SVM combine with species and genus data. (D) RF combine with species data. (E) RF combine with genus data. (F) RF combine with species and genus data. (G) DL combine with species data. (H) DL combine with genus data.


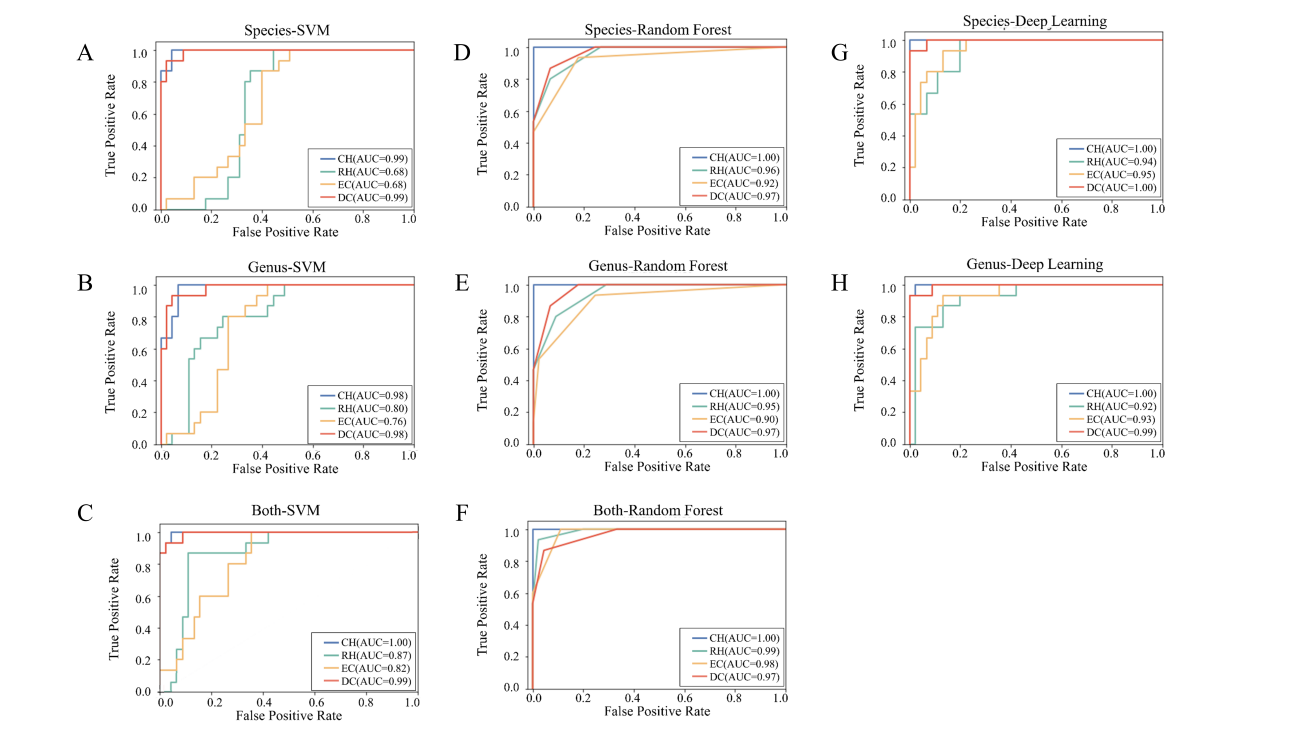
**Figure.S6 Comparison of receiver operating characteristics (ROC) of other eight models**

(A) SVM combine with species data. (B) SVM combine with genus data. (C) SVM combine with species and genus data. (D) RF combine with species data. (E) RF combine with genus data. (F) RF combine with species and genus data. (G) DL combine with species data. (H) DL combine with genus data.


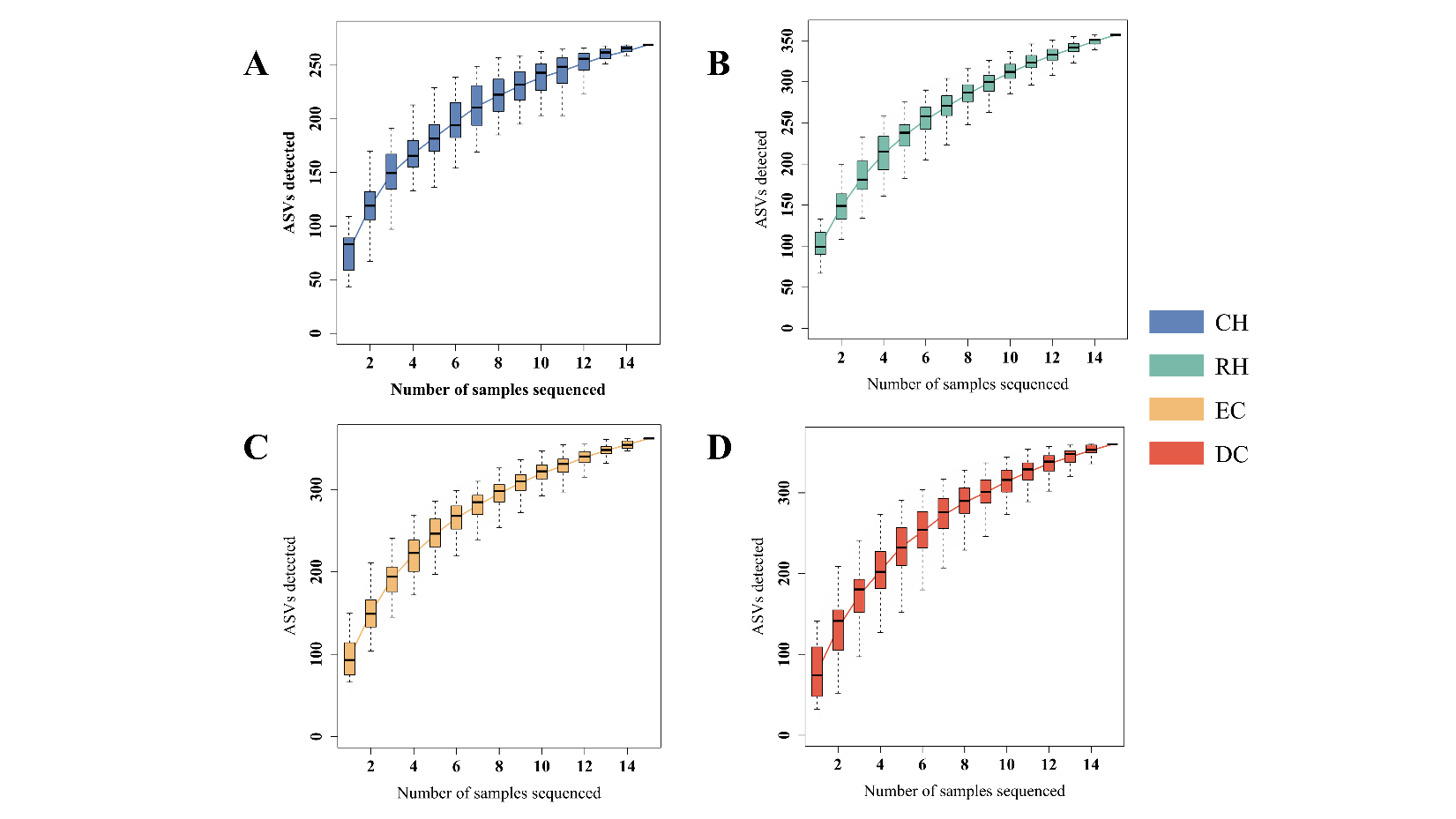


**Figure.S7 Rarefaction curves evaluating the relative abundance per sample**

AS the sample size reached 14, the rarefaction curves for each group generally approached saturation. (A) Rarefaction curves of detected species relative abundance of confident health (CH) group. (B) Rarefaction curves of detected species relative abundance of relative health (RH) group. (C) Rarefaction curves of detected species relative abundance of enamel caries (EC) group. (D) Rarefaction curves of detected species relative abundance of detin caries (DC) group.
